# Microvascular imaging in diffuse thyroid disease: diagnostic performance of subtraction ultra-micro angiography

**DOI:** 10.3389/fendo.2026.1892757

**Published:** 2026-10-27

**Authors:** Stefania Bunceanu, Andreea Bena, Octavian Constantin Neagoe, Roxana Pui, Diana Raluca Streinu, Marco Marian, Dana Stoian

**Affiliations:** 1 Department of Doctoral Studies, Victor Babes University of Medicine and Pharmacy, Timisoara, Romania; 2 Center for Advanced Ultrasound Evaluation, Dr. D Medical Center, Timisoara, Romania; 3 Center for Molecular Research in Nephrology and Vascular Disease, “Victor Babes” University of Medicine and Pharmacy, Timisoara, Romania; 4 Discipline of Endocrinology, Second Department of Internal Medicine, University of Medicine and Pharmacy “Victor Babes”, Timisoara, Romania; 5 1st Department of Surgery, Victor Babes University of Medicine and Pharmacy, Timisoara, Romania; 6 Second Clinic of General Surgery and Surgical Oncology, Emergency Clinical Municipal Hospital, Timisoara, Romania

**Keywords:** Graves’ disease, Hashimoto disease, microvessels, thyroid gland, ultrasonography, color doppler

## Abstract

**Background:**

Quantitative assessment of thyroid microvascularity may improve the ultrasound evaluation of diffuse autoimmune thyroid disease, particularly when conventional Doppler findings overlap. This prospective single-center study investigated whether subtraction Ultra-Micro Angiography (sUMA), quantified using the color pixel percentage (CPP) index, differentiates chronic autoimmune thyroiditis (CAT) from Graves’ disease (GD) and healthy thyroid parenchyma.

**Methods:**

A total of 179 participants were included: 84 controls, 74 with newly diagnosed, untreated CAT, and 21 with newly diagnosed, untreated GD. All participants underwent standardized B-mode ultrasonography followed by sUMA using the same ultrasound platform and linear transducer. Three CPP sUMA measurements were obtained in each thyroid lobe, and the lobe medians were averaged to obtain the whole-gland CPP sUMA value. Interlobar and group differences, correlations, multivariable associations, apparent ROC performance, bootstrap cut-off distributions, and repeated stratified five-fold internal validation were analyzed.

**Results:**

CPP sUMA values did not differ significantly between the right and left lobes. Mean CPP sUMA increased progressively from controls (23.25 ± 6.02%) to CAT (50.49 ± 13.58%) and GD (79.90 ± 8.86%). In CAT, CPP sUMA correlated positively with thyroid volume and TSH and inversely with FT4 and was significantly higher in overt than in subclinical disease. In GD, CPP sUMA correlated positively with FT3, FT4, and TSI. Differences between the diagnostic groups remained significant after adjustment for age, sex, BMI, and thyroid volume. Apparent ROC analysis showed high discrimination between CAT and controls (AUC 0.980; cut-off >31.55%; sensitivity 93.2%; specificity 90.5%) and between GD and CAT (AUC 0.966; cut-off >62.00%; sensitivity 100%; specificity 78.4%). GD and controls were completely separated in the analyzed cohort. During internal validation, mean accuracy was 88.5% for CAT versus controls, 82.0% for GD versus CAT, and 98.9% for GD versus controls.

**Conclusion:**

CPP sUMA provided a quantitative tissue-level estimate of thyroid microvascularity and maintained high discriminatory performance during internal validation. However, the proposed cut-offs are hypothesis-generating and require confirmation in larger multicenter cohorts using different ultrasound platforms. Once externally validated, CPP sUMA may complement thyroid function testing, antibody assays, and conventional ultrasound in the assessment of autoimmune diffuse thyroid disease.

## 1 Introduction

Ultrasonography is currently the reference imaging modality for thyroid evaluation, due to its high spatial resolution and its ability to distinguish normal from abnormal parenchyma with high accuracy in both focal and diffuse thyroid disease (DTD) (1). Beyond the morphologic information provided by grayscale imaging, the addition of Doppler-based techniques enables real-time assessment of parenchymal vascularity, thereby adding clinically relevant functional information in both healthy thyroid glands and DTD (2). This added value is particularly important in autoimmune DTD, most notably chronic autoimmune thyroiditis (CAT) and Graves’ disease (GD), in which vascular abnormalities may complement grayscale findings and improve diagnostic confidence when interpreted alongside clinical and biochemical data (3).

Thyroid vascularity reflects the interplay between trophic and immune-mediated regulation rather than circulating thyroid hormone levels alone (4). In CAT, vascular remodeling appears to be stage dependent: in the early phases, particularly before treatment initiation, elevated TSH levels and ongoing autoimmune inflammation may enhance vascular endothelial growth factor (VEGF) and fibroblast growth factor (FGF) driven angiogenic signaling and endothelial activation, promoting microvascular proliferation as a compensatory response aimed at maintaining oxygen delivery and residual thyroid hormone synthesis (5, 6). As the disease progresses, however, follicular destruction and fibrotic replacement of functioning parenchyma are likely to result in reduced or more heterogeneous vascularity (7). In GD, vascular changes are typically more pronounced. TSH receptor antibodies (TRAb) act as stimulatory antibodies against the TSH receptor, inducing sustained receptor activation and thereby promoting diffuse hyperfunction, gland enlargement, and marked hypervascularity (8, 9). The increase in thyroid blood flow appears to be driven predominantly by TSH-receptor stimulation and associated angiogenic activity, including increased VEGF expression and higher microvascular density in thyroid tissue (10).

Color Doppler (CD) ultrasonography represents the conventional ultrasound method for evaluating thyroid vascularity. In the setting of DTD, it has proven useful in the differential diagnosis of GD from other thyrotoxic states, with reported sensitivity and specificity of 88.9% and 87.5%, respectively, against scintigraphic findings (11). Accordingly, thyroid parenchymal vascularity can be assessed semi-quantitatively using the Schulz CD grading scale. However, the diagnostic value of this approach is limited by partial overlap between vascular patterns, particularly in early CAT, in which increased intraparenchymal flow may already be present, and in treated or less florid GD, where the classic hypervascular pattern may be less pronounced (2, 12). For this reason, Doppler vascular grading should be considered an adjunct rather than a standalone criterion in the assessment of DTD (3).

The principal limitation of conventional Doppler ultrasonography is its susceptibility to motion-related clutter artifacts, as the detected signal may originate not only from intravascular blood flow but also from adjacent tissue motion. Clutter-rejection filtering can reduce these artifacts; however, conventional wall filters often suppress true low-velocity Doppler signals as well, particularly those arising from small-caliber vessels (13). Moreover, the interpretation of Doppler vascular patterns remains partly operator dependent and subjective, which may affect reproducibility and diagnostic precision (14). Recent advances in microvascular ultrasound have partially overcome this limitation through adaptive, multidimensional filtering algorithms that more effectively separate slow blood flow from subtle surrounding tissue motion, thereby improving the depiction of microvascular perfusion (15).

Ultra-Micro Angiography (UMA) is an advanced Doppler ultrasound technique developed to facilitate the visualization of small-caliber vessels with low-velocity blood flow. It is based on ultrasound acquisitions using plane-wave and divergent-wave transmissions and incorporates an advanced wall-filtering/tissue-rejection algorithm that enables accurate differentiation between true microvascular flow and subtle motion in adjacent tissues, thereby substantially improving sensitivity for the detection of minute vessels (16). UMA comprises three modes which, although based on similar operational principles, are designed to highlight different hemodynamic parameters according to the examiner’s objective. Color UMA (cUMA) evaluates the velocity and direction of blood flow; power UMA (pUMA) assesses the intensity of the flow signal within the vessel lumen and may additionally provide directional information; and subtraction UMA (sUMA) further enhances the vascular features depicted by pUMA and is considered the most sensitive UMA mode for detecting very low-velocity blood flow. Moreover, UMA enables quantification of vascularization through the color pixel percentage (CPP) index, a parameter that provides a numerical percentage-based estimate of vascularity within the selected region of interest (17). However, the clinical utility of sUMA-derived quantitative vascularity assessment in autoimmune DTD remains insufficiently characterized, particularly regarding its ability to differentiate DTD from healthy thyroid parenchyma, distinguish GD from CAT, and identify associations between thyroid microvascularity and relevant biochemical, immunological, clinical, and sociodemographic parameters.

Considering this background, the present study investigated the clinical utility of sUMA for assessing thyroid vascularity in DTD. The primary objective was to determine whether sUMA-derived vascularity, quantified using the CPP index, can differentiate patients with autoimmune DTD from healthy controls and distinguish CAT from GD. The secondary objective was to examine the associations between sUMA-derived color pixel percentage (CPP sUMA) values and relevant biochemical, immunological, clinical, and sociodemographic parameters.

## 2 Materials and methods

### 2.1 Study cohort

This prospective single-center study was conducted over an 18-month period, from October 2024 to March 2026, at a specialized endocrine ultrasonography center in Timisoara, Romania.

During the study period, all individuals referred for thyroid ultrasound were screened consecutively, and 179 who met the eligibility criteria and provided informed consent were enrolled (**Figure 1**). No formal *a priori* sample-size calculation was performed; all eligible consenting participants enrolled consecutively during the predefined 18-month recruitment period were included.

**Figure 1:**
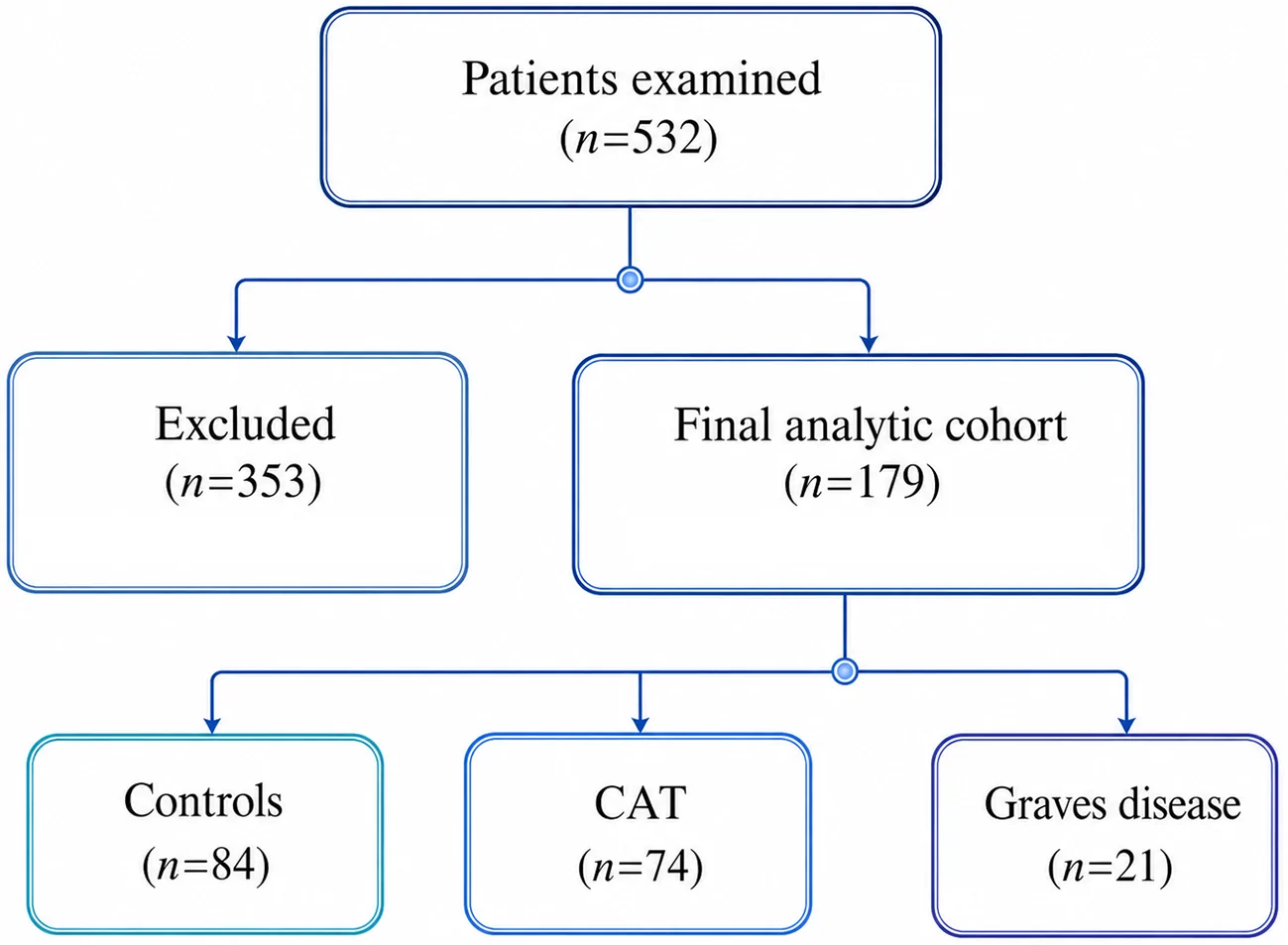
Flow diagram of participant selection and group classification.

The control group comprised individuals with no evidence of autoimmune thyroid disease, defined by normal thyroid ultrasound findings, serum TSH within the reference range, no history of thyroid disease, and negative thyroid autoantibodies. Laboratory interpretation was based on the following assay-specific reference intervals: TSH 0.27–4.50 µIU/mL, FT4 12–22 pmol/L, FT3 3.4–6.8 pmol/L, ATPO <34 IU/mL, ATG <115 IU/mL, and TSI <0.1 IU/L. Laboratory measurements were obtained within a maximum interval of 2 days before or after the sUMA examination. Both laboratory testing and the sUMA examination were completed before the initiation of any thyroid-specific treatment.

The second group included patients with newly diagnosed, untreated CAT who had either subclinical or overt hypothyroidism. The diagnosis was established in patients with serum TSH concentrations above the assay-specific reference range and evidence of thyroid autoimmunity, defined by elevated levels of anti-thyroid peroxidase antibodies (ATPO) and/or anti-thyroglobulin antibodies (ATG). Subclinical hypothyroidism was defined as an elevated TSH concentration with FT4 within the assay-specific reference range, whereas overt hypothyroidism was defined as an elevated TSH concentration with FT4 below the lower limit of the reference range. The third group consisted of patients with newly diagnosed, untreated GD, defined by suppressed serum TSH, elevated FT4 and/or FT3 concentrations, and positive thyroid-stimulating immunoglobulin (TSI) levels.

Patients were excluded if they had a history of head and neck irradiation, thyroid nodules detected on imaging, prior thyroid surgery, current or previous thyroid-specific treatment, or incomplete laboratory or imaging data. Conventional B-mode ultrasonography was used to exclude nodular disease in all participants and to confirm normal thyroid morphology in controls. CPP sUMA findings were not used for diagnostic classification. CAT and GD were classified according to the predefined biochemical and immunological criteria described above, whereas controls were defined using the laboratory criteria together with normal B-mode findings.

Written informed consent was obtained from all participants prior to enrollment. The study was conducted in accordance with the ethical principles of the Declaration of Helsinki and was approved by the Institutional Ethics Committee (Approval No. 92/01.10.2024, revised 2025).

### 2.2 Ultrasound examination

All participants underwent a multiparametric thyroid ultrasound examination performed by a single operator (A.B.) with more than 5 years of experience in thyroid ultrasonography. The protocol began with B-mode imaging, followed by sUMA.

The sUMA operator was blinded to the final laboratory-based diagnostic classification at the time of image acquisition and CPP sUMA measurement. Conventional B-mode findings required for eligibility assessment, including the absence of thyroid nodules and the presence or absence of diffuse parenchymal abnormalities, were recorded before sUMA activation. CPP sUMA measurements were subsequently performed according to the standardized acquisition protocol described below and were not used to assign patients to the diagnostic groups.

During the examination, patients were placed in the supine position with the neck extended, and ultrasound gel was applied to the anterior cervical region. All scans were obtained using the same ultrasound system (Mindray Resona R9, Mindray Bio-Medical Electronics Co., Ltd., Shenzhen, China) equipped with a 3–13 MHz linear transducer (L15-3WU), operated at 11 MHz.

Thyroid volume (TV) was calculated for each lobe using measurements obtained in three dimensions. In addition, the presence of nodules and the echogenicity of the thyroid parenchyma were systematically assessed on grayscale imaging. Only cases without nodular lesions were further evaluated by sUMA for microvascular analysis.

The same transducer was used for sUMA acquisition. In the longitudinal plane, the central and thickest portion of each thyroid lobe was identified on B-mode imaging, after which sUMA mode was activated. Upon activation, the system automatically displayed a larger sUMA imaging box, which was positioned over the thyroid lobe and adjusted so that its lateral dimension did not exceed 75% of the field of view. Gain was maintained at 40, and the wall filter was fixed at 46 Hz for all examinations.

Before quantitative image acquisition, patients were instructed to remain still while the probe was held motionless for 5–10 seconds to minimize motion-related artifacts. Within the larger sUMA imaging box, a smaller rectangular region of interest (ROI) measuring approximately 0.8–1.0 × 1.5–2.0 cm was manually positioned for CPP quantification (**Figure 2**). Its size was adapted to individual thyroid anatomy, but it was consistently placed in the same relative central region of each lobe and entirely within the thyroid parenchyma, deep to the anterior capsule without including it. The superior and inferior poles, major thyroid arteries, and all surrounding tissues were excluded from the quantitative ROI.

**Figure 2:**
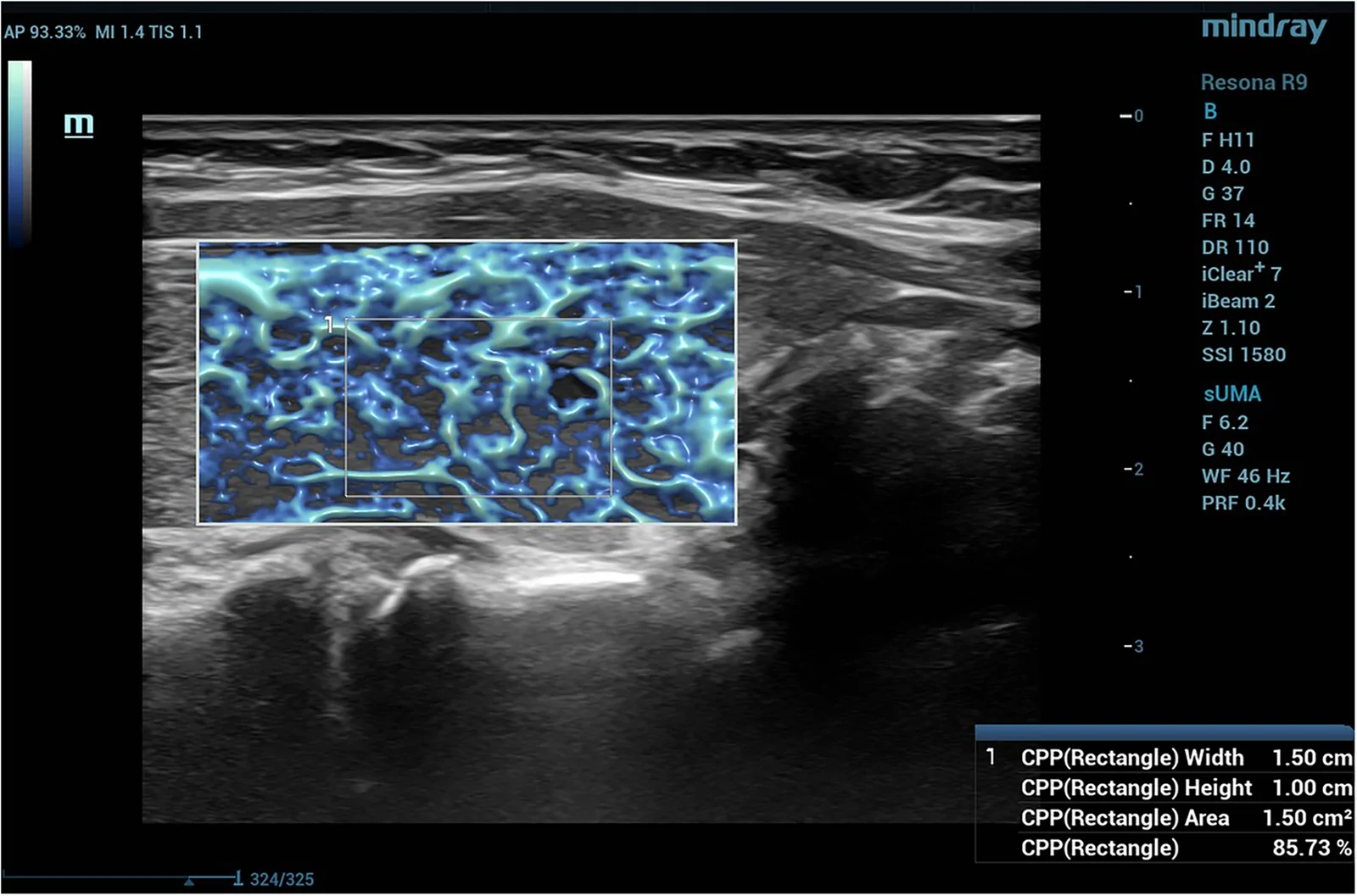
Quantitative assessment of thyroid microvascularity using sUMA in a patient with Graves’ disease. The grayscale B-mode image shows the central portion of a thyroid lobe in the longitudinal plane. The larger rectangular box contains the pseudocolour-coded sUMA overlay depicting intraparenchymal microvascular flow. The smaller central rectangle (ROI; 1.50 × 1.00 cm) represents the quantitative region of interest positioned entirely within the thyroid parenchyma. The CPP sUMA measured within this ROI was 85.73%. CPP, color pixel percentage; ROI, region of interest; sUMA, subtraction Ultra-Micro Angiography.

All repeated measurements were obtained during the same ultrasound examination. The left thyroid lobe was evaluated first, followed by the right lobe. For each lobe, three distinct sUMA images were acquired. For every acquisition, the same operator repositioned the quantitative ROI according to the standardized anatomical criteria described above, and one CPP measurement was recorded from each image. This resulted in three separate CPP measurements for each lobe. The median was calculated separately for the right and left lobes, and the mean of the two lobe-specific median values was used as the subject-level CPP sUMA value in the principal statistical analyses.

### 2.3 Statistical analysis

Statistical analyses were performed using MedCalc Statistical Software, version 23.5.5 (MedCalc Software Ltd., Ostend, Belgium). Python, version 3.12.13, was used for the multivariable analysis, resampling-based internal validation and repeatability analysis. For each thyroid lobe, three CPP sUMA measurements were obtained, and the median was used. Right- and left-lobe values were compared using the paired t-test. As no significant differences were found, their mean was used as the whole-gland CPP sUMA value.

An exploratory *post hoc* assessment of within-session intraobserver repeatability was performed using the three prospectively acquired CPP measurements from each thyroid lobe. Repeatability was evaluated separately for the right and left lobes and within each study group. Absolute-agreement, single-measure intraclass correlation coefficients were calculated using a two-way mixed-effects model and are presented with 95% confidence intervals. Within-subject coefficients of variation (CVw) were also calculated from the three repeated measurements. The analyses were performed separately within each diagnostic group to avoid inflation of the ICC estimates by the marked differences in CPP sUMA values between the study groups.

Normality was assessed using the Shapiro–Wilk test. Normally distributed variables are presented as mean ± standard deviation, and non-normally distributed variables as median and interquartile range. The three groups were compared using one-way analysis of variance or the Kruskal–Wallis test. Categorical variables were compared using the chi-square test or Fisher’s exact test. CPP sUMA values according to thyroid functional status in patients with CAT were compared using the independent-samples t-test. Correlations with continuous variables were assessed using Spearman’s coefficient, while the association with sex was assessed using point-biserial correlation. The Benjamini–Hochberg method was applied separately within each group to correct for multiple correlation analyses. Multivariable linear regression was performed using HC3 robust standard errors. Model 1 included diagnostic group, age, sex, and body mass index (BMI), while Model 2 additionally included thyroid volume. Thyroid function and antibody values were not included because they contributed to diagnostic classification and were not independent of the diagnostic groups.

Receiver operating characteristic (ROC) analysis was used to evaluate the diagnostic performance of CPP sUMA. Optimal cut-offs were identified using the Youden index. Area under the curve (AUC) confidence intervals and cut-off distributions were obtained from 2,000 stratified bootstrap resamples, while exact binomial confidence intervals were calculated for sensitivity, specificity, and accuracy. Internal validation was performed using 1,000 repetitions of stratified five-fold cross-validation. In each repetition, cut-offs were determined in the training sets and applied to the corresponding held-out data. Sensitivity, specificity, and accuracy were calculated from the combined held-out predictions, while AUC was averaged across the five held-out folds. A random seed of 2025 was used for all resampling analyses. Because the cut-offs were derived from the present dataset, they should be considered exploratory and require external validation.

## 3 Results

### 3.1 Baseline characteristics of the study population

**Table 1** summarizes the demographic, clinical, and biochemical characteristics of the control, CAT, and GD groups. No significant differences in age or sex distribution were observed among the three groups, whereas BMI differed significantly, with the lowest values observed in patients with GD.

**Table 1:** Baseline demographic, clinical, thyroid ultrasound, and laboratory characteristics of participants across the study groups.

Characteristic	Control (n=84)	CAT (n=74)	GD (n=21)	p-value
Age (years)	45 (31.50 – 56.00)	49.5 (33.00 – 61.00)	42 (32.25 – 54.25)	0.29
BMI (kg/m^2^)	26.60 (22.40 – 28.85)	25.75 ± 4.27	23.20 ± 3.51	0.01
TV (mL)	11 (9.30 – 13.65)	15.15 (12.76 – 17.70)	19.40 (17.30 – 23.80)	<0.001
TSH (µIU/mL)	2.2 (1.55 – 3.80)	8.3 (6.80 – 12.20)	0.005 (0.002 – 0.008)	<0.001
Free T4 (pmol/L)	16.5 ± 2.20	12.6 ± 2.58	42.3 (39.99 – 49.10)	<0.001
Free T3 (pmol/L)	–	–	13.01 ± 3.57	–
ATPO (IU/mL)	15.5 (10.00 – 22.50)	316.5 (162.00 – 751.00)	397 (167.25 – 1125.00)	<0.001
ATG (IU/mL)	16.5 (9.50 – 47.50)	44.5 (3.30 – 236.00)	198 (42.5 – 349.75)	<0.001
TSI (IU/L)	-	-	6.37 ± 3.53	-
Sex (Male/Female)	13 (15.5%)/71 (84.5%)	14 (18.9%)/60 (81.1%)	1 (4.8%)/20 (95.2%)	0.33

BMI, body mass index; TSH, thyroid-stimulating hormone; FT4, free thyroxine; ATPO, anti-thyroid peroxidase antibodies; ATG, anti-thyroglobulin antibodies; FT3, free triiodothyronine; TSI, thyroid-stimulating immunoglobulins; CAT, chronic autoimmune thyroiditis; GD, Graves’ disease; TV, thyroid volume.

TV showed a significant increase across the thyroid disease groups, particularly in patients with GD. Thyroid function parameters differed markedly among the analyzed groups. Patients with CAT had higher TSH and lower FT4 levels, consistent with reduced thyroid function, whereas patients with GD showed suppressed TSH and markedly increased FT4 levels. FT3 and TSI were measured only in patients with GD, with elevated FT3 levels and positive TSI values.

Thyroid autoantibody levels also differed significantly among groups. ATPO and ATG values were higher in patients with CAT and GD compared with controls.

### 3.2 Distribution of CPP sUMA values across study groups

A paired t-test was used to assess differences in vascularization between the right and left thyroid lobes within each of the three study groups. As shown in **Table 2**, no significant interlobar differences were observed in any group. Therefore, the mean CPP sUMA value of the two lobes was used in subsequent analyses as a representative measure of overall thyroid vascularization.

**Table 2:** Paired comparison of thyroid vascularization between the right and left lobes across the study groups.

Study group	CPP sUMA RTL, %	CPP sUMA LTL, %	p-value	Mean CPP sUMA, %	95% CI of mean, %
Control	22.743	23.765	0.256	23.254 ± 6.019	21.948–24.561
CAT	50.192	50.796	0.501	50.494 ± 13.575	47.348–53.639
GD	79.542	80.248	0.525	79.895 ± 8.859	75.863–83.928

CPP sUMA, color pixel percentage measured by sUMA; RTL, right thyroid lobe; LTL, left thyroid lobe; CAT, chronic autoimmune thyroiditis; GD, Graves’ disease; CI, confidence interval.

Whole-gland CPP sUMA values differed significantly among the three study groups (*p* < 0.001), as shown in **Figure 3**. The lowest values were observed in the control group, followed by patients with CAT, while patients with GD exhibited the highest values. A limited overlap was noted between the upper range of values in controls and the lower range observed in CAT, as well as between the highest values in CAT and the lowest values in GD. Notably, no overlap was observed between the control and GD groups, indicating a clear distinction in thyroid vascularization between these two subgroups.

**Figure 3:**
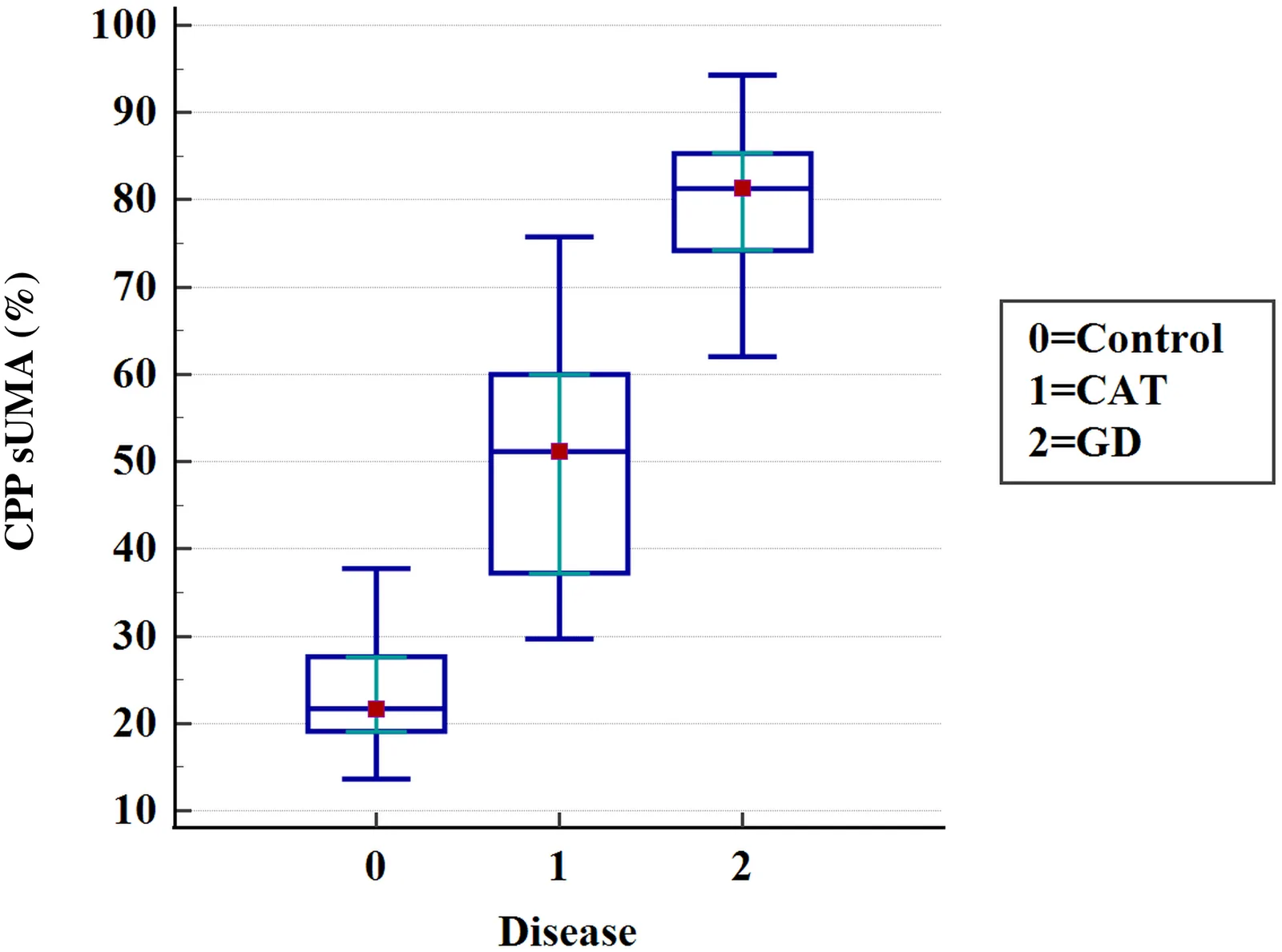
Distribution of CPP sUMA values across the three analyzed groups. Box-and-whisker plot showing CPP sUMA values in controls, patients with CAT, and patients with GD. For each group, the horizontal line inside the box represents the median; the box indicates the 25th–75th percentiles; and the whiskers represent values within 1.5×IQR of the quartiles. CPP sUMA, color pixel percentage measured by sUMA; CAT, chronic autoimmune thyroiditis; GD, Graves’ disease; IQR, interquartile range.

Within the CAT group, mean CPP sUMA values were significantly higher in patients with overt hypothyroidism than in those with subclinical hypothyroidism (60.26 ± 10.65% vs 44.20 ± 11.41%, *p* < 0.001), as shown in **Table 3**.

**Table 3:** CPP sUMA values according to thyroid functional status in patients with CAT.

Thyroid functional status	n	Mean CPP sUMA ± SD, %	95% CI	p-value
Subclinical hypothyroidism	45	44.20 ± 11.41	40.77–47.63	<0.001
Overt hypothyroidism	29	60.26 ± 10.65	56.20–64.31	

CPP sUMA, color pixel percentage measured by sUMA; CAT, chronic autoimmune thyroiditis; SD, standard deviation; CI, confidence interval.

### 3.3 Correlation analysis of CPP sUMA across study groups

**Table 4** summarizes the correlations between thyroid vascularization, assessed using CPP sUMA, and the clinical, demographic, ultrasound, and laboratory parameters analyzed within each study group. After subgroup-specific Benjamini–Hochberg adjustment, the only significant association in the control group was an inverse correlation between CPP sUMA and BMI, indicating that higher BMI was associated with lower measured thyroid vascularization values. An inverse relationship with BMI was also observed in the CAT and GD groups. However, in CAT, this association did not remain significant after adjustment for multiple testing, whereas it remained significant in GD.

**Table 4:** Correlation between CPP sUMA values and clinical, demographic, ultrasound and laboratory parameters in the analyzed groups.

Parameter	Group	Statistic	Sex	Age	BMI	TV	TSH	FT4	ATPO	ATG	FT3	TSI
CPP sUMA	Control	ρ/r_pb	0.04	-0.01	-0.51	0.07	0.12	-0.14	-0.04	0.04	-	-
		p	0.741	0.927	<0.001	0.511	0.276	0.199	0.704	0.730	-	-
		BH-adjusted q	0.847	0.927	<0.001	0.847	0.737	0.737	0.847	0.847	-	-
	CAT	ρ/r_pb	0.04	-0.04	-0.25	0.59	0.84	-0.65	0.22	0.04	-	-
		p	0.729	0.751	0.029	<0.001	<0.001	<0.001	0.066	0.724	-	-
		BH-adjusted q	0.751	0.751	0.058	<0.001	<0.001	<0.001	0.105	0.751	-	-
	GD	ρ/r_pb	-	0.03	-0.53	0.29	0.03	0.59	0.23	-0.03	0.61	0.80
		p	-	0.910	0.014	0.207	0.882	0.005	0.315	0.889	0.003	<0.001
		BH-adjusted q	-	0.910	0.031	0.373	0.910	0.015	0.473	0.910	0.015	<0.001

CPP sUMA, color pixel percentage measured by sUMA; ρ, Spearman’s rank correlation coefficient; r_pb, point-biserial correlation coefficient; BMI, body mass index; TSH, thyroid-stimulating hormone; FT4, free thyroxine; ATPO, anti-thyroid peroxidase antibodies; ATG, anti-thyroglobulin antibodies; FT3, free triiodothyronine; TSI, thyroid-stimulating immunoglobulins; TV, thyroid volume; CAT, chronic autoimmune thyroiditis; GD, Graves’ disease; BH, Benjamini–Hochberg; q, Benjamini–Hochberg-adjusted p-value.

In patients with CAT, CPP sUMA correlated positively with TSH and thyroid volume and inversely with FT4, suggesting an association between thyroid vascularization, gland volume, and thyroid functional status in this subgroup. In patients with GD, CPP sUMA correlated positively with FT3, FT4, and TSI and inversely with BMI, as also illustrated in **Figure 4**. These findings are consistent with relationships between thyroid vascularization, thyroid hormone levels, and autoimmune stimulation, although they do not establish causality or a specific vascular mechanism. Notably, no significant correlation between CPP sUMA and TSH was observed in GD. No other correlations remained significant after adjustment. The association with sex was not calculated in the GD group because only one male patient was included.

**Figure 4:**
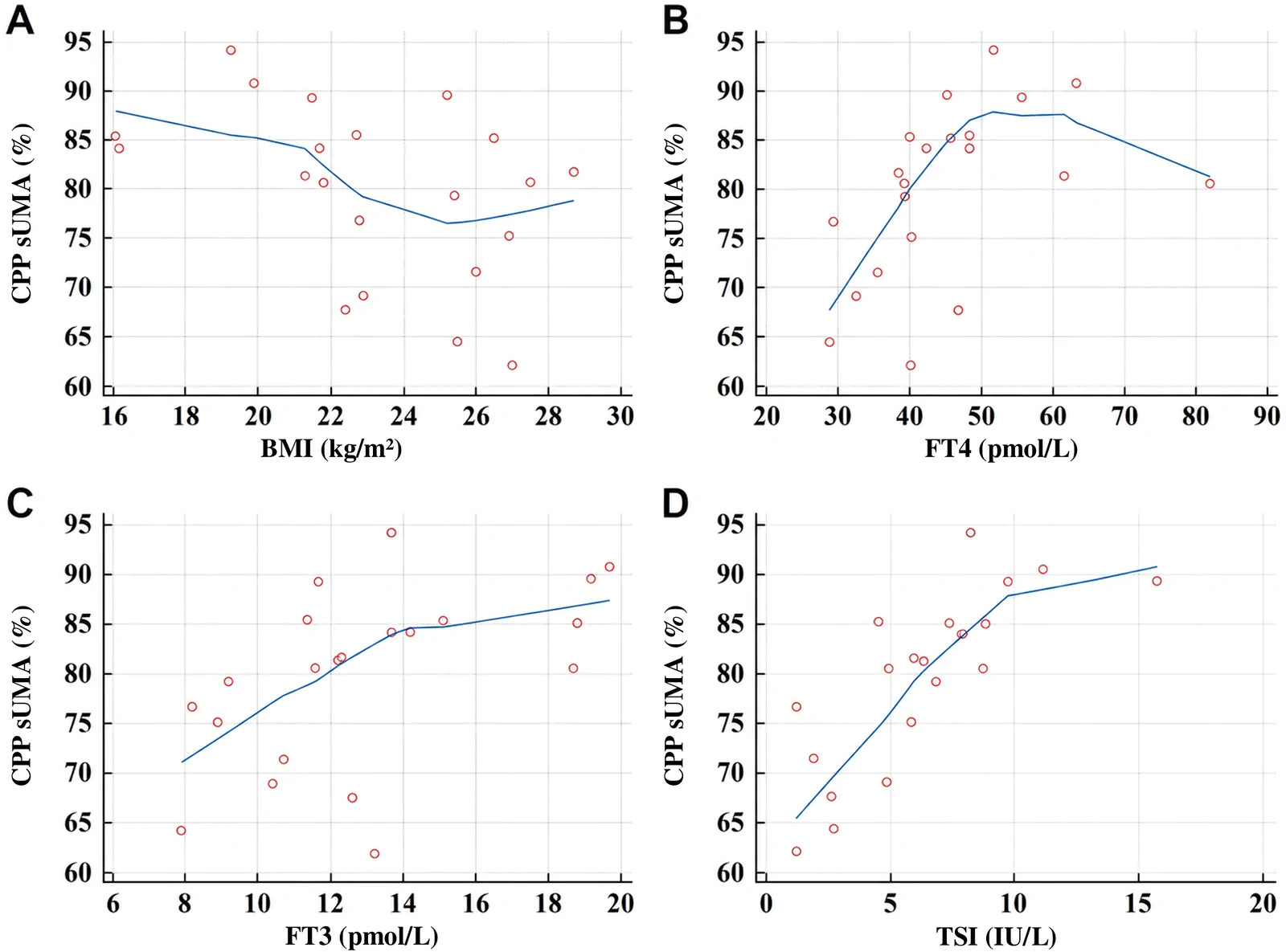
Correlation analysis of thyroid vascularization in patients with GD. Scatter plots showing the relationship between CPP sUMA values and **(A)** BMI, **(B)** FT4, **(C)** FT3, and **(D)** TSI in the Graves' disease group. Each circle represents an individual patient, and the fitted line illustrates the overall trend of the association. CPP sUMA, color pixel percentage measured by sUMA; BMI, body mass index; FT4, free thyroxine; FT3, free triiodothyronine; TSI, thyroid-stimulating immunoglobulins; GD, Graves’ disease.

### 3.4 Multivariable association with CPP sUMA

**Table 5** presents the results of the multivariable linear regression analysis. The models included diagnostic group, age, sex, and BMI, while Model 2 additionally included thyroid volume. Thyroid function and antibody values were not included because they formed part of the diagnostic criteria and were not independent of the diagnostic groups.

**Table 5:** Multivariable linear regression models of factors associated with CPP sUMA values.

Model	Term	Adjusted β	95% CI	P
Model 1	CAT vs control	26.33	23.11–29.55	<0.001
	GD vs control	54.17	50.28–58.06	<0.001
	GD vs CAT	27.84	23.13–32.55	<0.001
	Age	0.085	−0.003 to 0.172	0.058
	Female vs male	−1.89	−5.83 to 2.06	0.347
	BMI	−0.90	−1.21 to −0.59	<0.001
Model 2	CAT vs control	23.71	20.31–27.11	<0.001
	GD vs control	46.84	40.93–52.74	<0.001
	GD vs CAT	23.13	17.68–28.58	<0.001
	Age	0.055	−0.031 to 0.141	0.211
	Female vs male	−0.31	−4.31 to 3.68	0.877
	BMI	−0.83	−1.11 to −0.55	<0.001
	Thyroid volume	0.71	0.29–1.12	<0.001

CPP sUMA, color pixel percentage measured by sUMA; CAT, chronic autoimmune thyroiditis; GD, Graves’ disease; BMI, body mass index; CI, confidence interval. Model 1 adjusted for age, sex, and BMI; Model 2 additionally adjusted for thyroid volume. β represents adjusted differences in CPP sUMA percentage points or change per specified unit.

In Model 1, after adjustment for age, sex, and BMI, CPP sUMA was 26.33 percentage points higher in patients with CAT and 54.17 percentage points higher in patients with GD than in controls. CPP sUMA was also 27.84 percentage points higher in GD than in CAT. BMI was negatively associated with CPP sUMA, while age and sex showed no significant associations.

After thyroid volume was added in Model 2, the differences between the groups remained significant, although they were slightly reduced. CPP sUMA remained 23.71 percentage points higher in CAT than in controls, 46.84 percentage points higher in GD than in controls, and 23.13 percentage points higher in GD than in CAT. BMI remained negatively associated with CPP sUMA, while thyroid volume was positively associated with CPP sUMA. Age and sex remained non-significant. The adjusted R² increased from 0.805 to 0.826.

### 3.5 Diagnostic performance and internal validation of CPP sUMA for differentiating study groups

**Table 6** summarizes the apparent diagnostic performance of CPP sUMA across the study-group comparisons. CPP sUMA differentiated patients with CAT from controls with an AUC of 0.980 (bootstrap 95% CI, 0.963–0.992). At a cut-off of >31.55%, sensitivity was 93.2%, specificity was 90.5%, and accuracy was 91.8%. For differentiating GD from CAT, the AUC was 0.966 (bootstrap 95% CI, 0.930–0.992). A cut-off of >62.00% yielded 100% sensitivity, 78.4% specificity, and 83.2% accuracy. Complete separation was observed between GD and controls in the analyzed cohort, with an AUC of 1.0 and 100% apparent accuracy.

**Table 6:** Apparent diagnostic performance of CPP sUMA for differentiating the study groups.

Comparison	AUC (bootstrap 95% CI)	Cut-off (%)	Sensitivity (95% CI)	Specificity (95% CI)	Accuracy (95% CI)	TP/FP/TN/FN
CAT vs control	0.980 (0.963–0.992)	>31.55	93.2 (84.9–97.8)	90.5 (82.1–95.8)	91.8 (86.3–95.5)	69/8/76/5
GD vs CAT	0.966 (0.930–0.992)	>62.00	100 (83.9–100)	78.4 (67.3–87.1)	83.2 (74.1–90.1)	21/16/58/0
GD vs control	1.000 (1.000–1.000)	>37.62	100 (83.9–100)	100 (95.7–100)	100 (96.5–100)	21/0/84/0

CPP sUMA, color pixel percentage measured by sUMA; ROC, receiver operating characteristic; AUC, area under the ROC curve; CI, confidence interval; CAT, chronic autoimmune thyroiditis; GD, Graves’ disease; TP, true positive; FP, false positive; TN, true negative; FN, false negative. AUC 95% CIs were obtained from 2,000 stratified bootstrap resamples.

The bootstrap distributions of the optimal cut-off values are presented in **Table 7**. The median cut-offs were 31.47% for CAT versus controls, 66.44% for GD versus CAT, and 37.62% for GD versus controls.

**Table 7:** Bootstrap distribution of optimal CPP sUMA cut-off values across the study-group comparisons.

Comparison	Median cut-off (%)	IQR	2.5th–97.5th percentiles
CAT vs control	31.47	29.14–32.20	28.50–36.01
GD vs CAT	66.44	62.00–70.00	60.50–76.26
GD vs control	37.62	36.01–37.62	34.98–37.62

CPP sUMA, color pixel percentage measured by sUMA; IQR, interquartile range; CAT, chronic autoimmune thyroiditis; GD, Graves’ disease.

**Table 8** presents the results of repeated stratified five-fold internal validation. For CAT versus controls, mean sensitivity, specificity, and accuracy were 92.5%, 84.9%, and 88.5%, respectively. For GD versus CAT, the corresponding values were 82.4%, 81.8%, and 82%. GD and controls remained clearly separated, with mean sensitivity, specificity, and accuracy of 100%, 98.5%, and 98.9%, respectively. Although discriminatory performance remained high during internal validation, the identified cut-offs should be considered exploratory and require confirmation in independent cohorts.

**Table 8:** Diagnostic performance of CPP sUMA in repeated stratified five-fold internal validation.

Comparison	Sensitivity, % mean (2.5th–97.5th percentiles)	Specificity, % mean (2.5th–97.5th percentiles)	Accuracy, % mean (2.5th–97.5th percentiles)	Held-out AUC mean (2.5th–97.5th percentiles)	Training cut-off, % median (IQR)
CAT vs control	92.5 (86.5–95.9)	84.9 (82.1–88.1)	88.5 (86.1–90.5)	0.980 (0.974–0.987)	31.55 (29.14–31.55)
GD vs CAT	82.4 (71.4–90.5)	81.8 (77.0–87.8)	82.0 (78.9–85.3)	0.966 (0.950–0.981)	66.44 (62.00–66.89)
GD vs control	100 (100–100)	98.5 (96.4–98.8)	98.9 (97.1–99.0)	1.000 (1.000–1.000)	37.62 (37.62–37.62)

CPP sUMA, color pixel percentage measured by sUMA; AUC, area under the receiver operating characteristic curve; IQR, interquartile range; CAT, chronic autoimmune thyroiditis; GD, Graves’ disease.

### 3.6 Within-session intraobserver repeatability

Within-session intraobserver repeatability ranged from moderate to excellent across the study groups, as shown in **Table 9**. ICC(A,1) values ranged from 0.748 to 0.923. The highest agreement was observed in patients with CAT, while within-subject variability was lowest in patients with GD.

**Table 9:** Within-session intraobserver repeatability of CPP sUMA measurements.

Study group	Lobe	n	ICC(A,1) (95% CI)	CVw
Control	Right	84	0.765 (0.582–0.889)	13.3%
Control	Left	84	0.748 (0.556–0.880)	13.7%
CAT	Right	74	0.923 (0.847–0.966)	9.4%
CAT	Left	74	0.895 (0.795–0.953)	11.2%
GD	Right	21	0.841 (0.706–0.926)	4.5%
GD	Left	21	0.865 (0.745–0.938)	4.1%

ICC(A,1), two-way mixed-effects, absolute-agreement, single-measure intraclass correlation coefficient; CVw, within-subject coefficient of variation; CPP sUMA, color pixel percentage measured using sUMA; CAT, chronic autoimmune thyroiditis; GD, Graves’ disease; CI, confidence interval.

## 4 Discussion

Color Doppler (CD) ultrasonography has been used to assess thyroid vascularity for nearly four decades and remains the conventional technique in both healthy individuals and patients with thyroid disease (2, 18). By superimposing color-coded flow information onto the grayscale image, CD allows qualitative assessment of intrathyroidal vascularity and semiquantitative grading using the Schulz scale. However, its diagnostic value is limited by overlap in vascular patterns between healthy subjects and patients with thyroid disease and among diffuse thyroid disorders, particularly CAT and GD (19). A further limitation of CD is its reduced ability to distinguish slow flow in small-caliber intrathyroidal vessels from artifacts caused by adjacent tissue motion. Although conventional wall filters suppress these artifacts, they may also remove true low-velocity Doppler signals, leading to underestimation of subtle microvascular flow and reduced precision in the assessment of DTD (20).

Considering these limitations, recent advances in ultrasound technology have introduced several Doppler-derived microvascular imaging techniques that may refine the clinical evaluation of parenchymal vascularity. By improving the detection of slow flow within small-caliber intrathyroidal vessels and reducing motion-related artifacts, these methods may offer a more detailed assessment of vascular changes associated with DTD (13). These techniques include Superb Microvascular Imaging, Microvascular Flow Imaging, Angio Planewave UltraSensitive Doppler Imaging, and Ultra-Micro Angiography, the latter being the focus of the present study (21, 22).

Ultra-Micro Angiography extends this concept by integrating plane-wave and divergent-wave imaging with advanced spatio-temporal filtering. This combination enables effective suppression of tissue-motion artifacts while preserving weak Doppler signals generated by low-velocity flow in very small-caliber vessels (23). As a result, UMA provides a more sensitive depiction of thyroid parenchymal microvascularity than conventional CD imaging. Its cUMA, pUMA, and sUMA submodes provide complementary vascular information. Color and power UMA characterize flow direction and/or signal intensity, whereas sUMA reduces directional information to maximize sensitivity for fine vascular structures and slow microvascular flow, making it particularly useful for detecting subtle parenchymal vascular changes (24). In addition, sUMA permits objective quantification of microvascularity using the CPP index, which represents the proportion of color-coded pixels corresponding to Doppler-detectable blood-flow signals within the selected ROI, thereby providing a quantitative estimate of vascular signal density (22, 24).

To date, several studies have supported the clinical utility of sUMA in different medical fields. Its use has been investigated in inflammatory bowel disease, both for disease assessment and treatment-response monitoring (23), and it has also shown correlations with disease severity in rheumatoid arthritis (16). In addition, sUMA has been proposed as an adjunctive imaging tool in the diagnosis of lipedema (25) and biliary atresia (26). Moreover, encouraging results have been reported in the evaluation of suspicious nodular lesions in the breast, prostate, and thyroid, supporting its potential role in the characterization of microvascular changes associated with inflammatory and neoplastic disease (24, 27).

To the best of our knowledge, our research group was the first to establish CPP sUMA reference values for the normal thyroid gland (22). The present study extends this line of investigation by evaluating, for the first time, the potential role of sUMA in autoimmune DTD.

Our cohort included three groups of participants: healthy controls, patients with CAT, and patients with GD, with no significant differences among groups in terms of age or sex distribution.

The CPP sUMA results obtained in the control group are particularly relevant, as they provide the internal reference against which the findings in the thyroid disease groups should be interpreted. The mean thyroid CPP sUMA value in controls was 23.25 ± 6.02%, closely resembling that reported in our previously published study: 26.8% (18.8 – 36.2%) (22). This consistency was expected, given that the investigated population shared similar sociodemographic characteristics. Within the control group, no significant correlations were identified between CPP sUMA and age, sex, TSH, FT4, or TV, suggesting that, in healthy individuals, thyroid microvascularization remains relatively independent of these clinical, biochemical, and morphometric variables. In contrast, the only significant association observed was a moderate negative correlation between CPP sUMA and BMI (ρ = -0.51; q < 0.001). This finding may be explained, at least in part, by the technical influence of increased adipose tissue on ultrasound-based vascular assessment. In individuals with higher BMI, greater examination depth and beam attenuation may reduce the sensitivity for detecting low-velocity microvascular signals, leading to lower measured CPP sUMA values. A similar inverse association between BMI and thyroid vascularity was reported in a pediatric reference study using superb microvascular imaging, in which the thyroid vascularity index correlated negatively with BMI, while no significant correlations were observed with TSH or FT4. Although that cohort differs from the present adult population, these data support the need to consider BMI as a potential confounder when interpreting quantitative thyroid microvascular ultrasound measurements. Therefore, BMI should be taken into account in future studies aimed at validating CPP sUMA thresholds and may need to be incorporated into standardized acquisition protocols (28).

In the CAT group, the mean CPP sUMA was higher than in the control group, with a mean value of 50.49 ± 13.58%. This result is in line with previous studies reporting increased parenchymal vascularity in CAT using advanced Doppler-derived ultrasound techniques (7). However, these findings cannot be directly compared across studies because microvascular measurements are highly dependent on the ultrasound platform, signal-processing method, ROI selection, pulse repetition frequency, gain settings, and the algorithm used to calculate vascularity indices.

In patients with CAT, CPP sUMA correlated positively with TSH levels (ρ = 0.84, q < 0.001) and TV (ρ = 0.59; q < 0.001), while showing a strong inverse correlation with FT4 levels (ρ = −0.65; q < 0.001). In contrast, no significant associations were identified with age, sex, BMI, ATPO or ATG levels. Moreover, CPP sUMA was significantly higher in patients with CAT and overt hypothyroidism than in those with CAT and subclinical hypothyroidism (60.26% vs. 44.20%, p < 0.001). These associations are consistent with, but do not demonstrate, a relationship between thyroid microvascularity and functional or structural changes in CAT. Based on previous evidence, one possible explanation is that TSH-related trophic effects and autoimmune inflammation may be associated with endothelial activation and proangiogenic signaling, including VEGF-related pathways, which could contribute to increased thyroid vascularity and volume (6, 29). However, angiogenic mediators and histopathologic vascular remodeling were not directly assessed, and these correlations do not establish causality. For this reason, CPP sUMA may reflect thyroid functional status and gland structure in CAT, but it should not be considered direct evidence of a specific biological mechanism.

The GD group showed the highest CPP sUMA values, with a mean value of 79.90 ± 8.86%. Within this group, CPP sUMA correlated inversely with BMI (ρ = -0.53; q = 0.031) and positively with FT3 (ρ = 0.61; q = 0.015), FT4 (ρ = 0.59; q = 0.015) and TSI antibody levels (ρ = 0.80; q < 0.001). No significant correlations were observed with TSH, age, ATPO, ATG or TV. This correlation pattern appears consistent with the pathophysiology of clinically overt GD, which was present in all patients included in this group. Marked suppression of TSH may have limited its variability and therefore its association with CPP sUMA. In contrast, TSI reflects TSH receptor stimulation, the central mechanism underlying Graves’ hyperthyroidism and its associated increase in thyroid hormone synthesis and vascularity (30). The association between CPP sUMA and TSI levels suggests that thyroid microvascularity may vary with TSH receptor stimulation. However, because TSH receptor activity and the mechanisms underlying these vascular changes were not directly evaluated, this association does not demonstrate causality. The higher CPP sUMA values observed in GD than in CAT may be related to differences in thyroid stimulation between the two diseases, although this interpretation remains hypothetical (31).

The multivariable analysis showed that the differences between the diagnostic groups remained significant after adjustment for age, sex, BMI, and TV. In Model 2, CPP sUMA remained 23.71 percentage points higher in CAT than in controls, 46.84 percentage points higher in GD than in controls, and 23.13 percentage points higher in GD than in CAT. BMI remained negatively associated with CPP sUMA, while TV showed a positive association. Age and sex were not significantly associated with CPP sUMA. These findings indicate that the differences between the groups were not fully explained by the analyzed demographic and morphometric variables. Thyroid function and antibody values were not included because they formed part of the diagnostic criteria and were not independent of the diagnostic groups.

CPP sUMA showed high apparent diagnostic performance across the study-group comparisons. It differentiated CAT from controls with an AUC of 0.980 (95% CI, 0.963–0.992). At a cut-off of >31.55%, sensitivity was 93.2%, specificity was 90.5%, and accuracy was 91.8%. For differentiating GD from CAT, the AUC was 0.966 (95% CI, 0.930–0.992). A cut-off of >62.00% provided 100% sensitivity, 78.4% specificity, and 83.2% accuracy. Complete separation was observed between GD and controls in the analyzed cohort, with an AUC of 1.0 and 100% apparent accuracy at a cut-off of >37.62%. However, these results were obtained from the same dataset used to identify the optimal cut-offs and may therefore overestimate diagnostic performance, particularly considering the small number of patients with GD.

The bootstrap analysis yielded median cut-offs of 31.47% for CAT versus controls, 66.44% for GD versus CAT, and 37.62% for GD versus controls. The wider variation observed for GD versus CAT suggests that the exact threshold may depend on the characteristics of the analyzed sample. During repeated stratified five-fold internal validation, accuracy remained high for CAT versus controls and GD versus controls but was lower for GD versus CAT. These held-out results provide a more conservative estimate than the apparent analysis and show that some overestimation is likely when cut-offs are derived and tested in the same dataset. Therefore, the proposed thresholds should be considered hypothesis-generating and require confirmation in independent cohorts.

These observations are consistent with previous studies that evaluated thyroid microvascularization in DTD using other modern Doppler-based techniques. In thyrotoxic patients, microvascular ultrasonography distinguished GD from destructive thyroiditis with an AUC of approximately 0.852, a sensitivity of 87.0% and a specificity of 80.9% in an initial prospective study (31). A subsequent larger study identified the microvascular ultrasound vascularity index as a highly accurate ultrasound parameter, with a sensitivity of 79.6%, specificity of 84.3%, and an AUC of 0.856 (14). Although these values are not directly comparable with our results, the overall diagnostic concept is similar: increased thyroid vascularity provides relevant information for differentiating diffuse thyroid disorders. However, most previous comparative studies focused primarily on distinguishing GD from other forms of thyrotoxicosis rather than specifically evaluating CAT as a separate autoimmune thyroid disease group. Moreover, our study included only patients with newly diagnosed, untreated DTD, which may have contributed to more pronounced differences between the study groups.

To date, peak systolic velocity (PSV) remains the most extensively investigated and clinically used Doppler parameter for quantifying thyroid vascularization. This technique estimates thyroid blood flow by measuring velocities in the superior or inferior thyroid arteries. A diagnostic meta-analysis of superior thyroid artery PSV reported a pooled sensitivity of 0.86 (95% CI, 0.80–0.90), specificity of 0.93 (95% CI, 0.86–0.97), and AUC of 0.94 (95% CI, 0.92–0.96) for distinguishing GD from destructive thyroiditis (32). More recently, a meta-analysis including both superior and inferior thyroid artery velocities reported an SROC AUC of 0.91 for differentiating GD from destructive thyroid disorders and proposed a pooled PSV threshold of approximately 68.63 cm/s (95% CI, 59.12–78.13) for GD. However, the authors also emphasized the substantial heterogeneity among the included studies (33). Collectively, these data support the diagnostic relevance of thyroid hypervascularity in DTD. However, PSV reflects flow within feeding arteries rather than directly quantifying the microvascular signal within the thyroid parenchyma. By contrast, CPP sUMA measures vascular signal at the tissue level itself. This tissue-level assessment may be particularly relevant in autoimmune DTD, where alterations in parenchymal microvascularity may accompany disease-related inflammatory and functional changes.

Thyroid function tests and antibody assays remain the primary basis for diagnosing CAT and GD (29, 30). CPP sUMA should therefore be considered a complementary imaging parameter rather than an independent diagnostic test. In the present cohort, the proposed cut-offs showed promising discriminatory performance and may provide rapid additional quantitative information when laboratory findings are pending or equivocal or when scintigraphy is unavailable or contraindicated. However, these thresholds were derived from a selected population of untreated patients examined at a single center under standardized conditions, using the same ultrasound platform and operator. They may therefore represent an ideal-case scenario and should not be applied broadly without external validation. Their diagnostic performance may differ in treated, mild, or atypical disease and across ultrasound systems, acquisition settings, and clinical populations. Accordingly, the proposed cut-offs should be considered exploratory and should not replace standard biochemical and serological assessment.

Within this framework, sUMA may provide a quantitative estimate of thyroid vascularization, adding tissue-level vascular information to conventional clinical and imaging assessment. Its potential clinical role is therefore as an adjunctive quantitative layer in multiparametric thyroid evaluation that may support future standardization of vascular assessment.

The exploratory repeatability analysis showed moderate-to-excellent within-session agreement across the study groups, with ICC(A,1) values ranging from 0.748 to 0.923 and CVw values from 4.1% to 13.7%. Because each measurement was obtained from a different sUMA image and the ROI was repositioned for each acquisition, these findings reflect the combined repeatability of image acquisition and ROI placement within the same examination. The lower CVw values in GD may partly reflect the higher CPP sUMA values and the marked, diffuse thyroid hypervascularity, which may provide a more uniform vascular signal and reduce the effect of small differences in ROI placement. However, this explanation remains hypothetical because image-acquisition and ROI-placement variability were not assessed separately.

Several important limitations of the present study should be acknowledged. First, this was a single-center study, and all examinations were performed by one experienced operator using the same ultrasound system and transducer. Although this approach improved acquisition consistency, it limits the generalizability of the findings. Because all measurements were obtained using one ultrasound platform with fixed acquisition settings, the proposed CPP sUMA cut-offs should be considered machine-specific and cannot be directly transferred to other systems without appropriate calibration and external validation. CPP sUMA measurements may also be influenced by the transducer, acquisition settings, ROI placement, and tissue depth. Moreover, only within-session intraobserver repeatability was assessed; further studies involving separate examinations and additional operators are needed to evaluate between-session and interobserver reproducibility.

Second, the GD group was relatively small and included only 21 patients with newly diagnosed, untreated overt hyperthyroidism. Consequently, the apparent 100% sensitivity and complete separation between GD and controls should be interpreted with caution and may represent an ideal-case scenario. Only one male patient was included in the GD group, limiting sex-specific analyses. Similarly, the CAT group included untreated patients with subclinical or overt disease but did not include patients with advanced atrophic thyroiditis. The findings may therefore not apply directly to treated patients or to those with mild, atypical, or advanced disease, as treatment and disease stage may influence thyroid vascularity.

Third, the cut-offs were identified and initially evaluated using the same dataset. Although repeated stratified five-fold internal validation provided a more conservative estimate of diagnostic performance, it does not eliminate the optimism associated with the selected population, small GD group, and absence of an independent validation cohort. The proposed thresholds should therefore be considered hypothesis-generating and require confirmation in larger external cohorts.

In addition, none of the patients with CAT or GD was receiving thyroid-specific treatment at the time of examination. Although the inclusion of untreated patients reduced the influence of treatment-related changes in thyroid vascularity, it does not fully reflect routine clinical practice, where many patients are already receiving levothyroxine or antithyroid treatment. Because treatment may alter thyroid function, gland volume, and vascularity, the proposed CPP sUMA values and cut-offs may not apply directly to treated patients.

Taken together, these findings suggest that CPP sUMA may add quantitative information on thyroid microvascularity to multiparametric thyroid assessment, particularly when conventional Doppler patterns overlap. CPP sUMA should be considered a complementary parameter, and the cut-offs identified in this cohort should be regarded as exploratory. Future studies should include larger multicenter cohorts, independent external validation, between-session intraobserver and interobserver reproducibility analyses, different ultrasound platforms, and adjustment for tissue depth. Longitudinal studies including treated patients are also required to determine how CPP sUMA changes during treatment and follow-up.

## 5 Conclusion

In conclusion, CPP sUMA offers a quantitative approach to assessing thyroid parenchymal microvascularity in autoimmune DTD. Its stepwise increase across controls, CAT, and GD, together with its promising discriminatory performance, supports its potential as a complementary imaging parameter alongside standard biochemical, serological, and ultrasound assessment. Further multicenter studies across diverse patient populations and ultrasound systems should focus on external validation of the proposed thresholds, assessment of reproducibility, and clarification of the potential role of CPP sUMA in future diagnostic and monitoring strategies.

## Data Availability

The data supporting the findings of this study and the full study protocol are available from the corresponding author upon reasonable request.
